# The role of betel-quid chewing in smoking cessation among workers in Taiwan

**DOI:** 10.1186/1471-2458-14-755

**Published:** 2014-07-28

**Authors:** Fu-Li Chen, Peter Y Chen, Tao-Hsin Tung, Yu-Ching Huang, Min-Chien Tsai

**Affiliations:** Department of Public Health, College of Medicine, Fu Jen Catholic University, No.510, Jongjheng Rd., Sinjhuang, New Taipei city, Taiwan; Department of Psychology, Auburn University, Auburn, Alabama USA; Department of Medical Research and Education, Cheng-Hsin General Hospital, Taipei city, Taiwan

**Keywords:** Betel-quid chewing, Smoking cessation, Workers

## Abstract

**Background:**

Current smokers exhibit a higher rate of betel-quid chewing than non-smokers. However, little is known regarding the extent to which betel-quid chewing may affect attempts to quit smoking and smoking cessation. The aim of the present study is to examine the association between betel-quid chewing and patterns of quitting smoking. Specifically, we explore whether betel-quid chewing is associated with (1) current smokers who have never attempted to quit versus those who have attempted to quit and have failed, those who are in the process of quitting, and successful cessation smokers, and (2) current smokers who have attempted to quit and have failed versus those who have successfully quit smoking.

**Methods:**

A telephone survey of 7,215 workers was conducted and obtained an 88.6% response rate. In the survey, the respondents’ smoking and betel-quid chewing statuses were recorded and a list of covariates was assessed.

**Results:**

After controlling for the effect of the covariates, betel-quid chewing was found to be more highly associated with current smokers who have never attempted to quit, compared to current smokers who are in the process of quitting (OR = 12.72; 95% CI = 1.05–154.26), successful cessation smokers (OR = 3.62; 95% CI = 2.32–5.65), and smokers who have attempted to quit and have failed (OR = 1.37; 95% CI = 1.06–1.77), respectively. In addition, betel-quid chewing is more highly associated with a failure to quit smoking than with successfully quitting smoking (OR = 3.46; 95% CI = 2.17–5.51).

**Conclusion:**

The findings support four plausible reasons why betel-quid chewing may dissuade smokers from quitting. These reasons highlight additional avenues for potentially reducing the smoking population in workplaces, such as considering work contexts and social norms, and product sales in smoking-cessation campaigns.

## Background

Past research has shown that smoking is the primary cause of at least 30% of all cancer deaths in the United States, and approximately 80% of deaths from chronic obstructive pulmonary disease, and early cardiovascular disease and deaths [1]. In addition to its detrimental effects on health, smoking has shown adverse impacts on employee absenteeism in Taiwan, and a loss of productivity and an increase in the medical premium based on 200 Scottish workplaces [2, 3]. The impact of smoking on workers’ health and wellbeing is particularly significant in Taiwan, given that approximately 40.6% of male workers and 3.44% of female workers smoke [4], compared to 30.2% of males and 2.9% of females in the Taiwan’s overall population in 2011 [5]. Thus, the need to target workers in order to reduce the smoking population is obvious.

Empirical evidence has shown that not all smokers are willing to quit smoking. Of those who attempt to quit, some fail. Benowitz estimates that 80% of smokers who attempt to quit smoking relapse within the first month, and only 3% remain abstinent for at least six months [6]. According to a recent report by the Taiwan Bureau of Health Promotion [7], 39.3% of smokers have tried to quit, yet only 7.5% have succeeded in quitting (i.e., been abstinent for at least six months).

Past research has investigated factors associated with attempts to quit smoking and success in smoking cessation. These include perceived barriers to or self-efficacy in quitting smoking [8, 9], the use of nicotine patches [10], a low level of nicotine dependence [8], a low level of daily cigarette consumption [11], enforcing smoke-free laws and/or smoking restriction policies [12, 13], and implementing workplace smoking-cessation programs [14]. However, the role of betel-quid chewing in attempts to quit smoking and/or success in smoking cessation has rarely been studied.

Betel-quid is a combination of areca nut, betel leaf, and slaked lime. It is packed with flavoring ingredients such as condiments and sweetening agents. Betel-quid is considered the fourth most widely consumed addictive substance after caffeine, tobacco, and alcohol [15, 16], with 600 million people chewing betel-quid worldwide, particularly in Asia and Asian-migrant communities [17].

In contrast to some Asian countries, including Malaysia, Indonesia, Sri Lanka, and Nepal, betel-quid is mainly consumed without tobacco in Taiwan [18, 19]. According to the most recent national survey in Taiwan [20], the rate of betel-quid chewing is 7% (95% CI = 6.6–7.3), which represents approximately 1.61 million chewers. In Taiwan, betel-quid chewers tend to be male smokers, with low levels of income and education, who work in blue-collar industries [21].

Betel-quid chewing has been linked to oral cancer, esophageal cancers, obesity [22], periodontal disease [23], and cardiovascular disease [24]. Further, the adverse effects of betel-quid chewing on the aforementioned diseases tend to be exacerbated when chewers are also smokers. For instance, Wen et al. reveal that smokers who chew betel-quid exhibit a risk of oral cancer approximately three times higher than those who only smoke [25]. In general, current smokers exhibit a higher rate of betel-quid chewing (27.5%) than non-smokers (2.5%) [25], and those who quit smoking tend to stop chewing betel-quid as well [26]. Given the consistent findings about a positive relationship between these behaviors, it seems important, from both a practical and a scientific perspective, to examine whether betel-quid chewing plays a significant role in the process of smoking cessation.

From a practical perspective, it has been argued that betel-quid is not independent from smoking, given that the majority of betel-quid chewers are also smokers (approximately 92.6%) [25]. Thus, it is strategically important to know whether betel-quid plays an important role in attempts to quit smoking, as well as in the success of smoking cessation. From a scientific perspective, little is known about the extent to which betel-quid affects attempts to quit smoking and smoking cessation, even though the literature has generally shown a positive relationship between betel-quid chewing and cigarette smoking.

The aim of the present study is to investigate the association between betel-quid chewing and patterns of quitting smoking by extending previous research into factors that may facilitate or inhibit the cessation of smoking. Specifically, we examine whether betel-quid chewing is more closely associated with current smokers who have never attempted to quit (hereafter, the ‘no-attempt group’) than with smokers who have attempted to quit, including: (1) current smokers who have attempted to quit and have failed (hereafter, the ‘failed-attempt group’); (2) workers who are in the process of quitting (hereafter, the ‘quitting group’); and (3) former smokers (hereafter, the ‘successful-cessation group’). In addition, we assess whether betel-quid chewing is more closely associated with current smokers who have attempted to quit and have failed than with successful cessation smokers.

## Methods

### Respondents and procedure

A telephone survey, with an 88.6% response rate, was conducted by the Bureau of Health in Taoyuan County, Taiwan. Taoyuan County is 1,220 km^2^ in area and is located approximately 40 km southwest of Taipei in northern Taiwan. The survey participants consisted of 3,314 non-workers, and 7,215 workers, and non-workers were excluded in the study. The workers are from nine major occupations classified according to the third version of the International Standard Classification of Occupations (ISCO-88): professionals; senior officials and managers; technicians and clerks; salespersons and service workers; craft and related workers; plant and machine operators and assemblers; personal and protective services workers; elementary occupations; and skilled agricultural and fishery workers. Of the respondents, 5,251 (72.7%) were non-smokers or had smoked fewer than 100 cigarettes in their lives. Thus, they were excluded from the subsequent analyses. The remaining 1,964 respondents were retained in the analyses.

### Ethical considerations

This study received permission from and was reviewed by the Bureau of Health in Taoyuan County, Taiwan. The interviewers first explained to participants the purpose and voluntary nature of the study. All respondents were assured of the anonymity and confidentiality of their responses.

### Measures

#### Smoking status

**Figure 1:**
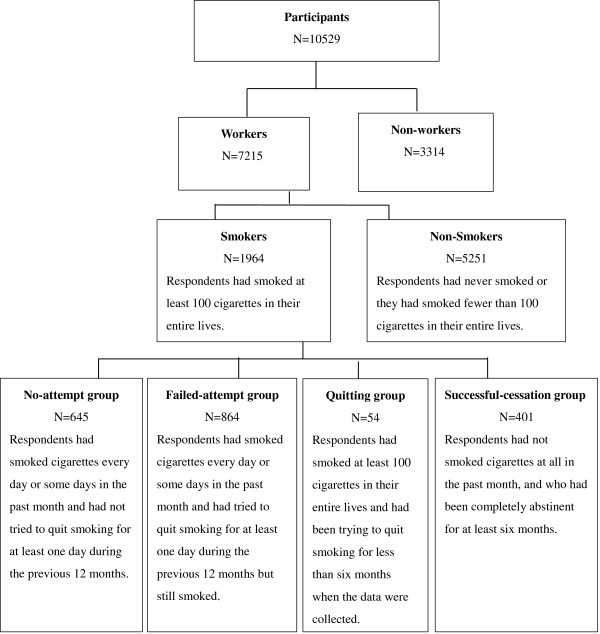
**Smokers classification flowchart.**

Four groups of respondents—current smokers who had never attempted to quit (the ‘no-attempt group’), current smokers who had attempted to quit and had failed (the ‘failed-attempt group’), smokers who were in the process of quitting (the ‘quitting group’), and former smokers (the ‘successful-cessation group’)—were created. A classification flowchart describing how these four groups were created is depicted in Figure 1.

First, to determine whether respondents are/were smokers, they were asked whether they had smoked at least 100 cigarettes in their entire lives [27, 28]. Those respondents who had smoked at least 100 cigarettes in their entire lives were asked whether they had smoked cigarettes “every day,” “some days,” or “not at all” in the past month. Respondents who had not smoked cigarettes at all in the past month, and who had been completely abstinent for at least six months, were classified as the *successful-cessation group* (N = 401) [27, 28]. Respondents who had smoked at least 100 cigarettes in their entire lives and had been trying to quit smoking for less than six months when the data were collected were classified as the *quitting group* (N = 54). Respondents who had smoked cigarettes every day or some days in the past month and had not tried to quit smoking for at least one day during the previous 12 months were classified as the *no-attempt group* (N = 645) [27]. Respondents who had smoked cigarettes every day or some days in the past month and had tried to quit smoking for at least one day during the previous 12 months but still smoked were classified as the *failed-attempt group* (N = 864).

#### Betel-quid chewing

Respondents were asked whether they had ever chewed betel-quid, with the following response categories: (1) I never chew betel-quid; (2) I have, but I have not chewed betel-quid at all in the past month; (3) I have chewed betel-quid some days in the past month; and (4) I have chewed betel-quid every day in the past month. Respondents were considered betel-quid chewers if they had chewed betel-quid some days or every day in the past month [25].

### Controlled variables

#### Demographic characteristics

Demographic variables include age, education (elementary school, high school, college or above), sex, and occupation.

#### Knowledge about tobacco hazards

Five true–false statements developed by Huang [29] were used to assess respondents’ knowledge about tobacco hazards. Sample statements included: “Smoking would adversely affect males’ fertility,” “People tend to get lung cancer if they smoke at an early age,” and “Smoking light cigarettes has less adverse impacts on health than smoking regular cigarettes”. The total score range was 0–5 points. The discrimination index for each item, computed according to the procedure outlined by Ebel [30], ranged from 0.37 to 0.72, with an average of 0.53, which demonstrates reasonable item discrimination to differentiate between people who do and do not possess the knowledge about tobacco hazards. Similarly, the item difficulty index for each item was calculated and ranged from 0.64 to 0.82, with an average of 0.73, which suggests that these items are relatively easy.

#### Attitude toward smoking

A five-item scale developed by Huang [29] was used to assess each respondent’s attitude toward smoking, with five response categories ranging from 1 (strongly disagree) to 5 (strongly agree). Item scores were reversed where required so that high scores reflected a negative attitude toward smoking. Sample items included: “Smoking is a good way to relieve stress,” and “Smoking is a personal matter, and it should not be restricted”. Cronbach’s alpha of the scale in the present study was 0.66.

#### Cigarette consumption

Cigarette consumption was assessed by asking respondents how many cigarettes they had smoked per day on average over the past 30 days.

#### Smoking restrictions at home and at work

Respondents were asked whether there were smoking restrictions at work, with three response choices: totally prohibited, banned in some areas, and no restriction. In addition, they were asked whether smoking was allowed inside their home, with three response choices: not allowed in the house, allowed in some areas, and no restriction.

#### Exposure to second-hand smoking at home and at work

Respondents were asked whether other people had smoked in front of them at home over the past week, and whether other people had smoked in front of them at work over the past week, with two response choices: no and yes.

### Data analysis

Hierarchical logistic regression was conducted to investigate whether betel-quid chewing is more closely associated with current smokers who have never attempted to quit than with those who have attempted to quit. To conduct the study, a list of variables related to workers who smoke were statistically controlled. These variables include age [31], education, sex [32], occupation [33], knowledge about tobacco hazards [12], attitude toward smoking [34], exposure to second-hand smoking at home and at work [35], smoking restrictions at home and at work [13], and cigarette consumption [11]. The final variable is not applicable to the successful-cessation group because cigarette consumption is zero by default. Specifically, smoking status (the no-attempt group vs. the failed-attempt group and the quitting group, respectively) was regressed on betel-quid chewing after controlling for age, education level, gender, occupation, knowledge about tobacco hazards, attitude toward smoking, exposure to second-hand smoking at home and at work, smoking restrictions at home and at work, and cigarette consumption. In addition, smoking status (the no-attempt group vs. the successful-cessation group) was regressed on betel-quid chewing after controlling for the effects of the above covariates except for cigarette consumption.

Similarly, hierarchical logistic regression was conducted to examine whether betel-quid chewing relates to the failure of smoking cessation by regressing smoking status (the failed-attempt group vs. the successful-cessation group) on betel-quid chewing after controlling for the effects of the above covariates except for cigarette consumption.

## Results

The results section consists of two parts. First, descriptive statistics of the studied variables by smoking status are presented in Table 1. Second, the results of hierarchical logistic regression are described in Tables 2 and 3.

**Table 1:** **Descriptive statistics of studied variables by smoking status**

	No attempt N (%)	Failed attempt N (%)	Quitting N (%)	Successful cessation N (%)	χ ^2^-test
**Gender**					21.72*
Female	88 (13.64)	85 (9.84)	10 (18.52)	76 (19.00)	
Male	557 (86.36)	779 (90.16)	44 (81.48)	324 (81.00)	
**Education level**					16.06*
Elementary school	37 (5.74)	54 (6.26)	2 (3.70)	40 (10.05)	
High school	390 (60.47)	516 (59.79)	26 (48.15)	210 (52.76)	
College above	218 (33.8)	293 (33.95)	26 (48.15)	148 (37.19)	
**Occupations**					15.44
Senior officials and managers	113 (17.52)	153 (17.71)	7 (12.96)	78 (19.50)	
Professionals	41 (6.36)	62 ( 7.18)	8 (14.81)	39 ( 9.75)	
Technicians & clerks	127 (19.69)	176 (20.37)	11 (20.37)	90 (22.50)	
Salespersons & service workers	163 (25.27)	196 (22.69)	15 (27.78)	83 (20.75)	
Skilled agricultural and fishery work, craft and related trades workers, and elementary occupations	201 (31.16)	277 (32.06)	13 (24.07)	110 (27.50)	
**Smoking restriction at home**					121.19*
Totally prohibited	46 (7.14)	93 (10.79)	11 (20.37)	116 (29.07)	
Banned in some areas	226 (35.09)	280 (32.48)	14 (25.93)	77 (19.30)	
No restriction	372 (57.76)	489 (56.73)	29 (53.7)	206 (51.63)	
**Smoking restriction at work**					48.82*
Totally prohibited	68 (10.79)	116 (13.54)	10 (18.52)	98 (24.87)	
Banned in some areas	257 (40.79)	383 (44.69)	28 (51.85)	148 (37.56)	
No restriction	305 (48.41)	358 (41.77)	16 (29.63)	148 (37.56)	
**Exposures to SHS at home**					67.92*
No	279 (44.29)	377 (44.25)	32 (60.38)	267 (67.09)	
Yes	351 (55.71)	475 (55.75)	21 (39.62)	131 (32.91)	
**Exposures to SHS at work**					30.67*
No	150 (23.66)	241 (28.12)	20 (38.46)	155 (39.04)	
Yes	484 (76.34)	616 (71.88)	32 (61.54)	242 (60.96)	
**Betel quid chewing**					105.99*
No	426 (66.25)	626 (72.54)	52 (96.30)	369 (92.25)	
Yes	217 (33.75)	237 (27.46)	2 (3.70)	31 (7.75)	
	Mean^a^ ± SD	Mean^b^ ± SD	Mean^c^ ± SD	Mean^d^ ± SD	Scheffe’s F test
**Age**	38.06 (11.4)	38.64 (10.96)	38.63 (14.21)	43.44 (10.88)	21.827* a < d, b < d, c < d
**Knowledge about tobacco hazards**	3.23 (1.32)	3.52 (1.21)	3.5 (1.3)	3.75 (1.24)	14.781* a < b, a < d, b < d
**Attitude toward smoking**	14.11 (2.19)	14.66 (2.12)	15.4 (2.05)	16.23 (2.23)	76.005* a < b, a < c, a < d, b < d
**Cigarette consumption**	17.67 (10.04)	16.88 (9.98)	0.94 (1.84)	-	440.934* a > c, b > c

Note: SHS = second-hand smoking. *p < 0.05. ^a^No-attempt group; ^b^Failed-attempt group**;** ^c^Quitting group; ^d^Successful-cessation group.

**Table 2:** **Logistic regression of no attempt to quit versus attempt to quit**

	No attempt vs. failed attempt		No attempt vs. quitting		No attempt group vs. successful cessation	
	OR	95% CI	OR	95% CI	OR	95% CI
**Gender**						
Female	(Reference)		(Reference)		(Reference)	
Male	0.64*	0.44-0.93	0.52	0.12-2.31	2.73*	1.88-3.97
**Age (yrs)**	0.99	0.98-1.00	0.95	0.90-1.00	0.96*	0.95-0.97
**Education level**						
College above	(Reference)		(Reference)		(Reference)	
Elementary school	0.68	0.36-1.29	16.0	0.28-901.71	0.76	0.40-1.43
High school	0.90	0.69-1.17	1.27	0.32-5.00	0.97	0.71-1.33
**Occupations**						
Professionals	(Reference)		(Reference)		(Reference)	
Senior officials and managers	1.21	0.72-2.03	18.72*	1.93-181.10	1.56	0.9-2.71
Technicians & clerks	1.11	0.67-1.84	5.52	0.79-38.56	1.01	0.59-1.73
Salespersons & service workers	1.22	0.74-2.01	0.77	0.10-5.99	1.55	0.89-2.69
Skilled agricultural and fishery work, craft and related trades workers, and elementary occupations	0.99	0.6-1.64	0.97	0.1-9.18	1.22	0.7-2.12
**Knowledge about tobacco hazards**	0.86*	0.78-0.95	0.90	0.54-1.49	0.88*	0.78-0.99
**Attitude toward smoking**	0.91*	0.86-0.96	0.79	0.6-1.05	0.73*	0.68-0.78
**Exposures to SHS at work**						
No	(Reference)		(Reference)		(Reference)	
Yes	1.11	0.83-1.48	0.68	0.18-2.56	1.00	0.73-1.36
**Exposures to SHS at home**						
No	(Reference)		(Reference)		(Reference)	
Yes	0.78	0.61-1.00	1.28	0.36-4.57	1.71*	1.27-2.31
**Smoking restriction at work**						
No restriction	(Reference)		(Reference)		(Reference)	
Totally prohibited	0.83	0.55-1.24	1.22	0.16-9.33	0.67	0.44-1.01
Banned in some areas	0.84	0.65-1.09	0.42	0.08-2.07	1.04	0.75-1.45
**Smoking restriction at home**						
No restriction	(Reference)		(Reference)		(Reference)	
Totally prohibited	0.72	0.47-1.11	1.53	0.3-7.85	0.46*	0.32-0.67
Banned in some areas	1.08	0.84-1.39	2.99	0.71-12.6	1.90*	1.36-2.67
**Cigarette consumption**	1.00	0.99-1.02	2.96*	1.94-4.52	NA	NA
**Betel quid chewing**						
No	(Reference)		(Reference)		(Reference)	
Yes	1.37*	1.06-1.77	12.72*	1.05-154.26	3.62*	2.32-5.65
−2log likelihood	1675.52*		87.23*		1336.02*	
Nagelkerke R^2^	0.06		0.78		0.33	

Note. NA = Not applicable. SHS = second-hand smoking. *p < 0.05.

**Table 3:** **Hierarchical logistic regression of failed attempt versus successful cessation**

	Model 1		Model 2	
	OR	95% CI	OR	95% CI
**Gender**				
Female	(Reference)		(Reference)	
Male	3.6*	2.37-5.47	3.23*	2.13-4.92
**Age (yrs)**	0.96*	0.94-0.97	0.96*	0.95-0.98
**Education level**				
College above	(Reference)		(Reference)	
Elementary school	0.94	0.49-1.81	0.95	0.48-1.85
High school	1.07	0.77-1.49	1.05	0.75-1.47
**Occupations**				
Professionals	(Reference)		(Reference)	
Senior officials and managers	1.34	0.75-2.42	1.27	0.7-2.3
Technicians & clerks	0.85	0.48-1.51	0.86	0.48-1.54
Salespersons & service workers	1.4	0.78-2.51	1.41	0.78-2.56
Skilled agricultural and fishery work, craft and related trades workers, and elementary occupations	1.09	0.61-1.97	0.98	0.54-1.79
**Knowledge about tobacco hazards**	0.92	0.8-1.04	0.92	0.81-1.05
**Attitude toward smoking**	0.74*	0.69-0.8	0.75*	0.7-0.8
**Exposures to SHS at work**				
No	(Reference)		(Reference)	
Yes	1.08	0.78-1.5	1.01	0.72-1.4
**Exposures to SHS at home**				
No	(Reference)		(Reference)	
Yes	2.01*	1.47-2.75	1.9*	1.38-2.62
**Smoking restriction at work**				
No restriction	(Reference)		(Reference)	
Totally prohibited	0.66	0.43-1.03	0.69	0.44-1.08
Ban in some areas	1.1	0.77-1.55	1.11	0.78-1.59
**Smoking restriction at home**				
No restriction	(Reference)		(Reference)	
Totally prohibited	0.54*	0.36-0.8	0.53*	0.36-0.8
Ban in some areas	1.86*	1.3-2.66	1.92*	1.34-2.76
**Betel quid chewing**				
No			(Reference)	
Yes			3.46*	2.17-5.51
−2log likelihood	1140.96*		1108.86*	
Nagelkerke R^2^	0.30		0.33	

Note. SHS = second-hand smoking.

*p < 0.05.

As seen in Table 1, there are significant differences between all the studied variables among the four different smoking statuses—the no-attempt group, the failed-attempt group, the quitting group, and the successful-cessation group—except for occupations. Although different occupations tend to have different levels of smoking consumption [33], attempts to quit smoking (i.e., no attempt, failed attempt, quitting, successfully quitting) are similar among occupations based on the current findings. The findings in Table 1 also show that higher percentages of workers in both the no-attempt group and the failed-attempt group chew betel-quid than those in both the quitting and the successful-cessation groups.

As seen in Table 2, after controlling for the effects of a list of covariates, betel-quid chewing was shown to relate consistently to current smokers who had never attempted to quit, in contrast to the failed-attempt group (χ^2^(1) = 5.62, p = 0.02), the quitting group (χ^2^(1) = 5.21, p = 0.02), and the successful-cessation group (χ^2^(1) = 39.73, p = 0.00). Specifically, the odds ratio of chewing betel-quid estimated that the odds for the no-attempt group was 1.37 times the failed-attempt group, 12.72 times the quitting group, and 3.62 times the successful-cessation group. The averages of the predictive values across the classes (i.e., the percentage correctly classified in the no-attempt group and the percentage correctly classified in the other groups) were 56.6%, 88.7%, and 64.1%, respectively. Overall, these results suggest that betel-quid chewing plays an important role in predicting whether or not smokers attempt to quit smoking.

The results in Table 2 show that males, younger workers, workers with a positive attitude toward tobacco, and workers with a poorer knowledge about tobacco hazards, tend not to attempt to quit. Further, in contrast to workers who experience no restrictions on smoking at home, respondents who are prohibited from smoking anywhere at home are more likely to attempt to quit smoking. However, the pattern of quitting is reversed for workers who are prohibited from smoking in some areas at home. In other words, respondents who are permitted to smoke in some areas at home are less likely to attempt to quit smoking, compared to those who are permitted to smoke anywhere at home.

The role of betel-quid chewing is further highlighted by the findings reported in Table 3. After controlling for the effects of the covariates, betel-quid chewing was found to be associated with the failure of smoking cessation (χ^2^(1) = 32.11, p = 0.00). The odds ratio of betel-quid chewing estimated the odds for the failed-attempt group to be 3.46 times the successful-cessation group. The average of the predictive values across the classes was 67.7%. These findings suggest that betel-quid chewing plays an important role in predicting whether smokers will fail in attempting to quit smoking.

Table 3 displays similar results to those found in Table 2. Specifically, males, younger workers, and workers with a positive attitude toward tobacco are more likely to fail in their attempts to quit smoking. In addition, those who are exposed to second-hand smoking at home are more likely to fail to quit. Compared to those who experience no restrictions on smoking at home, respondents who are prohibited from smoking anywhere at home are more likely to quit smoking successfully. Again, the pattern of quitting is reversed for those who are prohibited from smoking in some areas at home. In other words, respondents who are permitted to smoke in some areas at home are more likely to fail to quit smoking, compared to those who are permitted to smoke anywhere at home.

## Discussion

Overall, after a list of related covariates are statistically controlled, the findings reveal that betel-quid chewing is more closely linked to current smokers who have never attempt to quit, compared to current smokers who have attempted to quit but have failed, smokers who are in the process of quitting, and smokers who have successfully ceased smoking. In addition, betel-quid chewing is more closely associated with a failure to quit smoking compared to success in quitting smoking.

A plausible interpretation of these findings is that smokers chew betel-quid as a means of quitting smoking. This is an intriguing proposition, although we could not identify evidence to suggest that betel-quid is chewed as a means to quitting smoking. Based on the data of the present study, the following means were used by participants in their attempts to quit smoking: self-control (90.5%); support from family members and friends (4.7%); medical or counseling services (2.7%); smoking-cessation education (0.6%); quit smoking hotline (0.4%); and others (1.1%), including acupuncture, exercise, diet, and prayer.

In addition to the above explanation, there are four plausible reasons, which are not mutually exclusive, why betel-quid may adversely influence the process of quitting smoking. It is well known that areca nut (part of betel-quid) contains psychoactive alkaloids, such as arecoline, which stimulate the central nervous system by, for example, increasing pupil dilation, skin temperature, pulse rate, and systolic blood pressure [36, 37]. These adaptive mechanisms are core biological components of a dependence syndrome. In cigarettes, it is nicotine, rather than psychoactive alkaloids, that induces stimulation and pleasure sensation [6]. Although the biological mechanism of dependence for betel-quid chewing and smoking are different, both behaviors tend to assist the user’s concentration and relaxation [6, 38]. These common stimulant-like effects may inadvertently “bond” both behaviors through conditioning and reinforcement. This bond may explain why betel-quid chewing is a major barrier in the process of quitting smoking. In addition, some betel-quid users may not be willing or interested in quitting smoking. Although it is no entirely clear why betel-quid users may not be interested or motivated to quit smoking, cultural background and social norms may play important roles in the process of quitting smoking.

Both betel-quid and cigarettes play an important role in fostering social interactions in certain sub-cultures. For instance, offering betel-quid, in addition to cigarettes, at wedding ceremonies or festivals in aboriginal Taiwanese communities is a way of showing respect to guests, and is deemed an important cultural ritual [39]. However, it is not the custom to offer betel-quid to guests among Hans people in Taiwan, although cigarettes may be offered at these occasions. This may explain why aborigines in Taiwan tend to smoke and chew betel-quid more often than Hans people [40].

In a similar vein, consumption of both betel-quid and cigarettes at work may be influenced by work contexts specific to certain occupations. Chuang, Chang, and Chang found that male blue-collar workers, particularly in transportation, construction, and fishing, tend to chew betel-quid and smoke to cope with the harsh working conditions (e.g., long hours and physically demanding work) [41]. In addition, the majority of tasks in these occupations are conducted outdoors, where smoking is always permitted, meaning that workers who smoke and chew betel-quid may be less inclined to change their behaviors, even though there is a smoke-free policy (indoors) at work. The social norms of chewing betel-quid and smoking to foster interactions among peers in these occupations also play an important role in reducing attempts to quit smoking.

Typically, betel-quid vendors in Taiwan also sell cigarettes, yet cigarette vendors (e.g., convenience/grocery stores and supermarkets) tend not to sell betel-quid. The sales outlets may pose greater challenges for workers who smoke and chew betel-quid than for those who only smoke, since cigarettes are readily available from betel-quid stalls.

## Conclusion

The present study was conducted by a cross-sectional design and the survey results rely on self-report. These limitations limit us from making any causal conclusion, and these limitations can be improved in future studies by utilizing a longitudinal design with multiple data sources. In general, the findings support the idea that betel-quid consumption plays a critical role in preventing smokers from quitting. The explanations presented in the discussion section suggest additional avenues for reducing the smoking population in workplaces, such as by considering work contexts specific to certain occupations, social norms for fostering interactions in certain sub-cultures, and the sale of cigarettes in betel-quid stalls. Government agencies in Taiwan, such as the Bureau of Health Promotion and the Department of Health, may consolidate efforts and budgets to reduce both behaviors by integrating tobacco control and betel-quid control policies and strategies, which are currently independently implemented. Presently, smoking-cessation services in Taiwan are provided through outpatient services in medical institutions and toll-free smoking-cessation counseling as well as social media campaigns. However, efforts to reduce betel-quid chewing are made through different media campaigns and oral cancer screening. Considering that betel-quid chewing may adversely affect users’ attempts to quit smoking, targeting both behaviors in health-promotion strategies seems cost-effective and efficient.
